# Leveraging Community Health Workers and a Responsive Digital Health System to Improve Vaccination Coverage and Timeliness in Resource-Limited Settings: Protocol for a Cluster Randomized Type 1 Effectiveness-Implementation Hybrid Study

**DOI:** 10.2196/52523

**Published:** 2024-01-12

**Authors:** Lavanya Vasudevan, Jan Ostermann, Nathan Thielman, Joy Noel Baumgartner, David Solomon, Anna Mosses, Amy Hobbie, Nicole L Hair, Chen Liang, Marco van Zwetselaar, Sayoki Mfinanga, Esther Ngadaya

**Affiliations:** 1 Hubert Department of Global Health Rollins School of Public Health Emory University Atlanta, GA United States; 2 Duke Global Health Institute Duke University Durham, NC United States; 3 Department of Health Services Policy & Management Arnold School of Public Health University of South Carolina Columbia, SC United States; 4 Department of Medicine Duke University Durham, NC United States; 5 School of Social Work University of North Carolina at Chapel Hill Chapel Hill, NC United States; 6 National Institute for Medical Research Muhimbili Research Centre Dar es Salaam United Republic of Tanzania; 7 Zwets IT Harskamp Netherlands

**Keywords:** childhood vaccinations, timeliness, vaccine hesitancy, digital health, community health workers, Tanzania, low- and middle-income countries, SMS, reminder, conditional incentive

## Abstract

**Background:**

Tanzania is 1 of 20 countries where the majority of unvaccinated and undervaccinated children reside. Prior research identified substantial rural-urban disparities in the coverage and timeliness of childhood vaccinations in Tanzania, with children in rural settings being more likely to receive delayed or no vaccinations. Further research is necessary to identify effective and scalable interventions that can bridge rural-urban gaps in childhood vaccination while accounting for multifaceted barriers to vaccination.

**Objective:**

This protocol describes a type 1 effectiveness-implementation hybrid study to evaluate Chanjo Kwa Wakati (*timely vaccination* in Kiswahili), a community-based digital health intervention to improve vaccination timeliness. The intervention combines human resources (community health workers), low-cost digital strategies (electronic communication, digital case management, and task automation), a vaccination knowledge intervention, and insights from behavioral economics (reminders and incentives) to promote timely childhood vaccinations.

**Methods:**

The study will be conducted in 2 predominantly rural regions in Tanzania with large numbers of unvaccinated or undervaccinated children: Shinyanga and Mwanza. Forty rural health facilities and their catchment areas (*clusters*) will be randomized to an early or delayed onset study arm. From each cluster, 3 cohorts of mother-child dyads (1 retrospective cohort and 2 prospective cohorts) will be enrolled in the study. The timeliness and coverage of all vaccinations recommended during the first year of life will be observed for 1200 children (n=600, 50% intervention group children and n=600, 50% nonintervention group children). The primary effectiveness outcome will be the timeliness of the third dose of the pentavalent vaccine (Penta3). Quantitative surveys, vaccination records, study logs, fidelity checklists, and qualitative interviews with mothers and key informants will inform the 5 constructs of the reach, effectiveness, adoption, implementation, and maintenance (RE-AIM) framework. The results will be used to develop an implementation blueprint to guide future adaptations and scale-up of Chanjo Kwa Wakati.

**Results:**

The study was funded in August 2022. Data collection is expected to last from February 2024 to July 2027.

**Conclusions:**

This study will address the lack of rigorous evidence on the effectiveness of community-based digital health interventions for promoting vaccination coverage and timeliness among children from sub-Saharan Africa and identify potential implementation strategies to facilitate the deployment of vaccination promotion interventions in low- and middle-income countries.

**Trial Registration:**

ClinicalTrials.gov NCT06024317; https://www.clinicaltrials.gov/study/NCT06024317

**International Registered Report Identifier (IRRID):**

PRR1-10.2196/52523

## Introduction

### Background

Globally, the number of children missing their first dose of the diphtheria-pertussis-tetanus (DPT) vaccine rose from 19 million in 2019 to 25 million in 2021 [1,2]. The vast majority (14.2 million, 78%) of such *zero-dose* children reside in 20 countries, including Tanzania [3]. As in other low- and middle-income countries (LMICs), substantial rural-urban disparities in routine childhood vaccination exist in Tanzania, with vaccination rates being lower in rural areas than in urban areas [4]. Using data from the 2015-2016 Tanzanian Demographic and Health Survey, we demonstrated gaps in vaccination coverage (receipt of each recommended vaccine dose by age 1 y) and timeliness (receipt of each vaccine dose within 28 d of the recommended age) for Tanzanian children [4]. The coverage of the first dose of the pentavalent vaccine (Penta1, which includes antigens of DPT, *Hemophilus influenzae*, and hepatitis B) was 79.4% nationally in this analysis, with the remaining 21% of children classified as zero dose [4]. We documented receipt of the third dose of the pentavalent vaccine (Penta3) in even fewer children (72.7%), suggesting dropouts in the multidose vaccine series. Finally, rural children had lower timeliness of vaccination (47.8% delayed for Penta3) than urban children (24.2% delayed for Penta3). New interventions are needed to reduce the number of children who are zero dose, receive delayed vaccines, or drop out and to close the rural-urban gap in vaccination [4,5]. Such interventions must consider multifaceted barriers to vaccination and variations in the availability of human resources and infrastructure in rural areas.

In prior research in southern Tanzania, we identified challenges with the availability of, and access to, vaccination services, including challenges with distance and transportation to health facilities, temporary nonavailability of vaccines owing to a lack of reliable refrigeration at health facilities, and vaccine wastage policies that prevented the use of multidose vials when clinics had low volumes of children to be vaccinated [6]. Service unreliability and lack of communication about service interruptions were noted as causes of frustration among mothers of vaccine-eligible children [6]. Our research also identified vaccine hesitancy owing to vaccine-related knowledge gaps and concerns. In general, rural mothers reported more vaccine-related knowledge gaps and concerns than urban mothers [6]. Despite challenges, vaccination intention was high among mothers, and conditional incentives were identified as a potential *nudge* to increase vaccination timeliness [7,8]. In addition, a community health worker (CHW)–delivered knowledge intervention was piloted and determined to be feasible to implement in rural areas. On the basis of the findings of this formative research and other published reports [9], we designed Chanjo Kwa Wakati (*timely vaccination* in Kiswahili, the most commonly spoken language in Tanzania), a community-based digital health intervention to improve childhood vaccination coverage and timeliness. The intervention seeks to combine human resources (CHWs), low-cost digital strategies (electronic communication, digital case management, and process automation), a vaccination knowledge intervention (counseling scripts addressing specific knowledge gaps), and insights from behavioral economics (reminders and incentives) to promote timely childhood vaccination.

### Objectives

This protocol describes our planned evaluation of the implementation and impact of Chanjo Kwa Wakati. The study seeks to contribute evidence to the literature in 3 key ways. First, although many studies have evaluated mobile phone–based reminders for promoting childhood vaccinations [10], evidence is lacking [9] for more complex community-based digital health interventions that target multifaceted barriers to vaccinations, such as those identified in our prior research [4,6,7]. Second, few community-based digital health interventions for promoting childhood vaccination have been evaluated using rigorous randomized controlled study designs, especially in sub-Saharan African countries and in rural areas [11-15]. Third, evidence on implementation strategies associated with deploying community-based digital health interventions in LMICs is limited but critically important for supporting scale-up in the context of highly resource-constrained national health systems. To bridge these gaps in the literature, we will use a type 1 effectiveness-implementation hybrid study [16,17] to evaluate the effectiveness of Chanjo Kwa Wakati in improving the timeliness of childhood vaccinations in rural areas in Tanzania and identify strategies that support its implementation. The detailed protocol of the study is presented in the following sections.

## Methods

### Study Overview and Aims

This type 1 effectiveness-implementation hybrid study [16,17] uses a cluster randomized trial to evaluate intervention effectiveness and mixed methods to describe implementation outcomes. A cluster randomized design was chosen because the delivery of the intervention is organized at the cluster level. In addition, there are ethical concerns with the randomization of intervention activities, including incentives, at the individual level in rural communities in Tanzania. The evaluation of the Chanjo Kwa Wakati intervention will be guided by the reach, effectiveness, adoption, implementation, and maintenance (RE-AIM) framework. The methods used in this study are described in accordance with the CONSORT (Consolidated Standards of Reporting Trials) checklist for cluster randomized studies (Multimedia Appendix 1). The study aims and hypotheses are described in the following subsections.

#### Aim 1

The first aim is to evaluate the effectiveness of Chanjo Kwa Wakati for increasing the timeliness of childhood vaccinations due by age 1 year compared with the standard of care. The effectiveness of Chanjo Kwa Wakati will be evaluated in a cluster randomized trial with 1200 mother-child dyads enrolled from the catchment areas of 40 rural health facilities (*clusters*) in 2 regions in Tanzania. Clusters will be randomized into an early or delayed onset study arm. The intervention will target the mother-child dyad; outcomes will be assessed at the child level. Our hypothesis is that Chanjo Kwa Wakati is effective for increasing the timeliness of childhood vaccinations due by age 1 year compared with the standard of care. The primary outcome measure used to evaluate intervention effectiveness will be a continuous measure of the timeliness of the third dose of the pentavalent vaccine (Penta3), due at age 14 weeks, and determined using the birth and vaccination dates abstracted from official vaccination cards issued for the child. A secondary outcome will be a binary measure of timeliness, defined as receipt of Penta3 within 28 days of the due date. Other outcomes measures include the timeliness and coverage of all other vaccine doses due by age 1 year.

#### Aim 2

The second aim is to evaluate the implementation factors associated with variation in intervention effectiveness and develop an implementation blueprint for intervention scale-up to other settings. Study logs, fidelity checklists, quantitative surveys, and qualitative interviews with mothers and key informants will be used to inform other constructs of the RE-AIM framework, specifically reach, adoption, implementation, and maintenance. Two key implementation outcomes of interest are participants’ reports of the receipt of intervention components (intervention fidelity) and their reports of intervention acceptability. Analyses of variation in intervention implementation at the cluster level and systematic variation in the effectiveness for aim 1 outcomes across children will guide the future optimization of Chanjo Kwa Wakati. The results will be used to develop an implementation blueprint to guide future adaptations and scale-up of Chanjo Kwa Wakati.

### Study Setting

The study will be conducted in 4 rural districts in Mwanza and Shinyanga regions in Tanzania. Both regions have large numbers of unvaccinated or undervaccinated children. The populations of Mwanza and Shinyanga region were estimated to be 3,699,872 and 2,241,299, respectively [18]. In 2022, only 56% of children aged 12 to 23 months in Mwanza region and 32.2% of children in Shinyanga region were estimated to have received all basic vaccinations, including one dose of Bacillus Calmette-Guerin vaccine, 3 doses of polio vaccine, 3 doses of DPT-containing vaccine, and one dose of measles-containing vaccine [19]. Fewer children aged 12-23 month were considered fully vaccinated according to the national vaccination schedule shown in Table 1 (32.8% in Mwanza and 2.5% in Shinyanga) [19]. Notably, 3.3% of children aged 12-23 months in Mwanza and 14.5% in Shinyanga were reported to have received no vaccinations. [19].

**Table 1:** Routine childhood immunizations recommended in Tanzania before age 1 year.

Antigen	Age at vax due date
Bacillus Calmette-Guerin (BCG) and oral polio vaccine (OPV) 0	At birth or first contact
OPV1; pentavalent vaccine comprising antigens of diphtheria, pertussis, tetanus, *Hemophilus influenza* B, and hepatitis B (Penta) 1; pneumococcal vaccine (PCV) 1; and rotavirus vaccine (Rota) 1	6 weeks
OPV2, Penta2, PCV2, and Rota2	10 weeks
OPV3, Penta3, PCV3, and injectable polio vaccine (IPV)	14 weeks
Measles and rubella 1	9 months

### Intervention Components

Chanjo Kwa Wakati focuses on individual-level barriers to timely vaccination to complement the Tanzanian government’s efforts to strengthen vaccination systems and reduce structural barriers [20]. Chanjo Kwa Wakati comprises the following individual-level intervention components: a vaccination knowledge intervention, reminders about upcoming vaccination due dates, service notifications, and conditional incentives for timely vaccination (Table 2). The intervention’s target population is the mother-child dyad from the time of pregnancy (third trimester) until the child is aged 1 year. Intervention activities are targeted toward the mother, whereas the timeliness of Penta3 (primary outcome) and other vaccinations recommended before age 1 year (secondary outcomes; Table 1) is assessed for the child.

**Table 2:** The Chanjo Kwa Wakati intervention.

Timing	Activities	Detailed description of activities
Last trimester of pregnancy	Enrollment Registration in digital health system Knowledge intervention Baseline assessment^a^	Informed consent^a^ Register pregnancy and phone numbers in the digital health system Identify knowledge gaps for an individualized knowledge intervention based on a study by Vasudevan et al [6] Assess postintervention change in vaccination knowledge and attitudes as well as potential correlates of coverage and timeliness^a^
≤4 wk after the date of delivery	CHW^b^ phone call or home visit	Ascertain date and place of birth as well as the receipt of vaccinations due at birth Register the birth, record the 6-wk vaccination due date, and update phone numbers in the digital health system
Before each vaccination due date (6, 10, and 14 wk, and 9 mo)	SMS text message reminders with incentive offers Backup: CHW phone calls or home visits Knowledge intervention	Messages are sent to the mother and vaccine advocates 7 d and 1 d before each vaccination Messages are individualized and account for information on service interruptions Messages include conditional incentive offers Individualized knowledge intervention focused on vaccinations due at an upcoming visit
≤1 wk after each vaccination due date	CHW phone call or home visit	Verify vaccination status and date against vaccination card and issue incentive as appropriate Update vaccination due dates (and thus reminders) as necessary
12-15 mo after birth	Follow-up assessment^a^	Validate vaccination coverage and dates using vaccination cards^a^ Issue incentive as appropriate

^a^Research activity. All other activities are intervention activities.

^b^CHW: community health worker.

#### Individualized Knowledge Intervention

The knowledge intervention consists of 9 true or false statements that assess key knowledge gaps about childhood vaccinations. A brief counseling script is provided with each statement that will be read out by the CHW after an incorrect response. Statements are based on prior research in Tanzania [6]. Statements and counseling scripts were tested extensively for content, comprehension, and cultural appropriateness with national key informants from Tanzania’s Immunization and Vaccines Development program, regional and district health officials, CHWs, and pregnant women from rural Tanzania. In addition to the in-person individualized vaccination education provided by CHWs, SMS text message–based vaccination promotion messages, sent via the Chanjo Kwa Wakati digital health system, will be individualized to each participant’s knowledge gaps and aligned with the child’s vaccination schedule.

#### Vaccination Reminder Messages

CHWs will verify the due date for the next vaccination using the child’s vaccination card and update the child’s information in the Chanjo Kwa Wakati digital health system. The system will automatically schedule SMS text message reminders to be sent 7 days and 1 day before each vaccination due date. The SMS text messages will include the name of the intended recipient, the child’s name, the vaccination due date, and other relevant information.

#### Service Notifications

In the event of stockouts or service nonavailability, SMS text messages with relevant information will be sent to mothers whose children are due for vaccination.

#### Incentive Offers

Conditional incentive offers will be specific to each child’s vaccination schedule. In our prior work [7,8], we identified a set of incentives (eg, pharmacy vouchers and birth certificates) that are likely to be acceptable to mothers. The specific incentives, their value, conditionality, and the timing of disbursement will be determined in discussion with local key informants.

### Implementation Strategies

Relevant implementation strategies will be identified in collaboration with local key informants, and a detailed logic model will be developed to guide study activities.

### Intervention Activities

**Figure 1:**
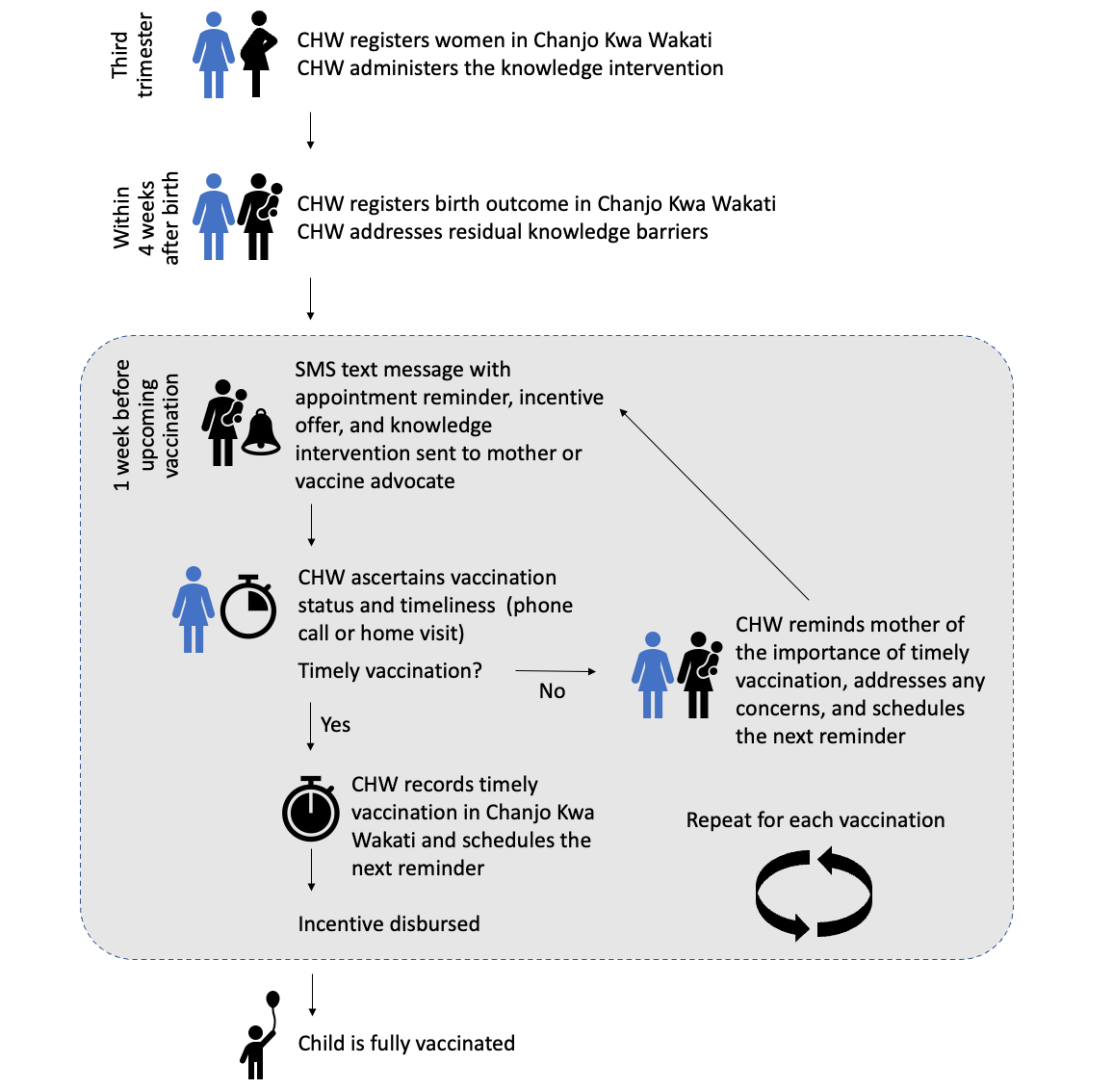
Sequence of intervention activities. CHW: community health worker.

Figure 1 describes the sequence of intervention activities. Pregnant women, enrolled in their third trimester, will receive a home visit from a CHW before the birth of their child, be registered with the Chanjo Kwa Wakati digital health system, and receive an individualized knowledge intervention to screen and counsel for common knowledge gaps and concerns about routine childhood vaccinations. After the birth of the child, the mother will receive individualized SMS text message reminders for upcoming vaccinations, messages aimed at mitigating persistent vaccination knowledge gaps, service notifications (eg, related to stockouts or service nonavailability), and conditional economic incentive offers aimed at encouraging timely vaccinations. CHWs will use the digital health system for work planning, recording vaccination dates and due dates, and following up on missed vaccination appointments and backstop the digital health system with phone calls and in-person visits as needed for vaccination reminders and assessing and addressing mothers’ knowledge gaps.

### Study Population

#### Sampling Area

The sampling area comprises 2 rural districts in Mwanza region and 2 rural districts in Shinyanga region, selected based on district-level information about vaccination rates, logistical considerations, and input from key informants.

#### Cluster Eligibility

Twenty clusters will be selected in each region for a total of 40 clusters. Clusters are defined as the catchment area of eligible health facilities in the sampling area. Eligible health facilities include public and public-designated (private not-for-profit facilities that serve the functions of public health facilities) hospitals, health centers, and dispensaries. Facilities must be operational, offer routine prenatal care and childhood vaccination services, have at least 2 active CHWs operating in the catchment area, and have reported at least 100 pregnancies or births in the year before study implementation. Eligibility will be determined initially using administrative data obtained from district offices and verified using surveys with health facilities.

#### Study Participants, Eligibility, and Recruitment

The study population will include 3 groups of participants.

##### Cross-Sectional Retrospective Cohort

This cohort (n=400) will be used for retrospective assessments of children’s vaccination records during their first year of life. Eligible participants will be mothers or legal guardians (henceforth referred to as *mothers*) of children aged 12 to 23 months, aged ≥15 years, and residing in the sampling area since the birth of the child.

Before recruiting this cohort, CHWs, with help from local key informants (eg, village leaders, health care providers, and traditional birth attendants), will compile a list of children aged 12 to 23 months residing in each cluster. The lists will be randomized, and mothers will be approached by the research team for eligibility determination, informed consent, and enrollment until 10 mother-child dyads from each cluster are enrolled into the study. Up to 40 additional women, who are not necessarily living in the study area but meet the other eligibility criteria, may be enrolled to pilot-test the study instruments.

##### Longitudinal Prospective Cohort

This cohort (n=800) will be used for prospective assessments of children’s vaccination records during their first year of life. Eligible participants will be pregnant women in their last trimester of pregnancy, aged ≥15 years, residing in the sampling area, and expected to reside in the sampling area until the child reaches age 1 year.

Before recruiting this cohort, CHWs, with help from local key informants (eg, village leaders, health care providers, and traditional birth attendants) will compile a list of pregnant women residing in each cluster. The lists will be randomized, and pregnant women will be approached by the research team for eligibility determination, informed consent, and enrollment until 10 pregnant women from each cluster are enrolled into the study. One additional eligible woman will be enrolled from each cluster during each prospective enrollment round to account for loss to follow-up (refer to the *Retention* subsection).

##### Participants in Qualitative Feedback

This group will be used to obtain feedback on the RE-AIM constructs. Eligible participants will be key informants at the national, regional, or local levels (target: n=12), health providers from participating facilities who are responsible for childhood vaccinations (target: n=40, approximately 1/cluster), CHWs in study clusters (target: n=80, approximately 2/cluster), and women enrolled in the prospective cohort (target: n=60).

To recruit these participants in qualitative work, a combination of purposive and snowball sampling strategies will be used. The numbers of participants represent target numbers; additional participants will be enrolled if saturation is not reached. Key informants, including policy makers, decision makers, and implementers at the national, regional, or local levels (eg, officials from Tanzania’s Immunization and Vaccines Development program as well as regional, district, and local health officials) will be asked to participate in qualitative interviews on implementation factors and to recommend other individuals who can provide relevant information. Health providers who are responsible for childhood vaccinations will be enrolled from participating health facilities. CHWs will be enrolled from participating clusters. Women will be purposively selected from the prospective cohort based on data collected during enrollment or follow-up surveys, study logs, or the digital health system during study implementation.

### Study Design

#### Cluster Randomized Trial Design

**Figure 2:**
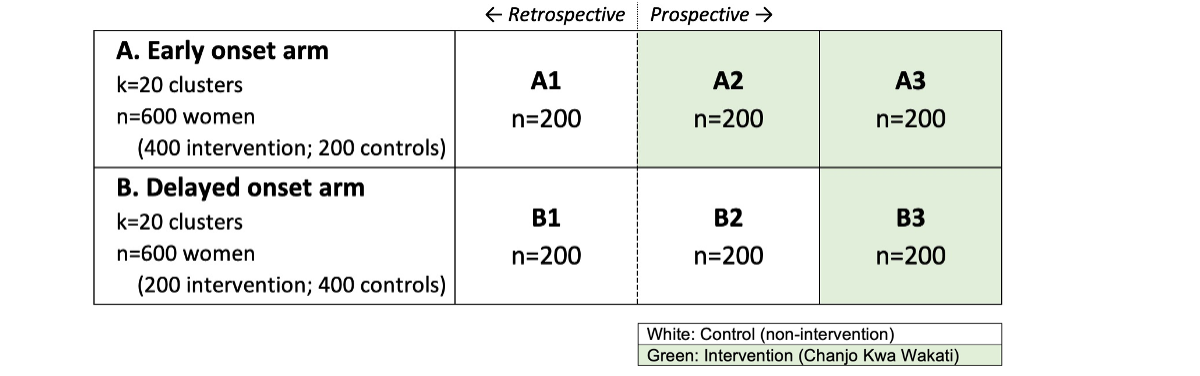
Design of the 2-arm cluster randomized trial (n=1200).

Forty rural health facilities and their catchment areas (*clusters*), 20 per region, will be randomized to an (A) early or (B) delayed onset study arm (Figure 2). From each cluster, 3 cohorts of 10 mother-child dyads (1 retrospective cohort and 2 prospective cohorts) will be enrolled into the study. For the retrospective cohort, 400 mothers of children aged 12 to 23 months (10/cluster) will be enrolled as a cross-sectional sample for retrospective assessments of children’s vaccination records during the first year of life. These cohorts, denoted by A1 and B1, will be part of the care-as-usual control group. For the longitudinal prospective cohort, 800 pregnant women in their last trimester of pregnancy will be enrolled during 2 rounds of enrollment (10 women/enrollment round/cluster) for prospective assessments of their children’s vaccination records during the first year of life. These cohorts are denoted by A2, A3, B2, and B3. In the first round of enrollment, 200 (50%) of the 400 prospective participants (those enrolled from early onset clusters; A2) will receive the intervention, and the other 200 (50%) participants (B2) will be part of the care-as-usual control group. In the second round of enrollment, all 400 participants (A3 and B3) will receive the intervention. In total, vaccination uptake and timeliness will be observed for 1200 children (n=600, 50% intervention group children and n=600, 50% nonintervention group children) during their first year of life. The nonintervention group children represent the *care-as-usual* control group in the trial.

The trial design, which analytically resembles a difference-in-difference design, allows for an analysis of the effectiveness of the intervention even in the context of contemporaneous changes in the rate and timeliness of vaccinations at the national level (eg, through national vaccination campaigns or changes in the vaccination schedule).

#### Evaluation Framework

The evaluation of the Chanjo Kwa Wakati intervention will be guided by the RE-AIM framework. Quantitative survey data will be the primary source of information for the analysis of the effectiveness of the intervention. These data will be complemented by study logs, fidelity checklists, and qualitative interviews with Tanzanian key informants, facility-based health workers, CHWs, and mothers to assess the acceptability, reach, and fidelity of Chanjo Kwa Wakati, as well as factors that may inform sustainability and scalability in the future.

### Outcomes Assessments

Vaccination outcomes are described in Table 3. Vaccination coverage and dates will be ascertained for each study child using government-issued vaccination cards, which will be photographed, scanned, or copied and entered by research staff. For the retrospective cohort, the enrollment survey will assess vaccination outcomes for the child; their participation will end after the enrollment survey. Outcome data for the prospective cohort will be collected during an endline survey 12 to 15 months after the birth of the child. All vaccination doses of all vaccination series will be tracked. For women who cannot provide vaccination cards at the time of the survey, consent will be sought to access their children’s paper-based or electronic vaccination records at their local health facility.

**Table 3:** Outcome measures for the cluster randomized controlled trial.

Outcome measure	Type	Source
Timeliness of the third dose of the pentavalent vaccine, Penta3 (number of days delayed)	Primary	Vaccination cards
Timeliness of Penta3 (≤28 days delayed)	Secondary	Vaccination cards
Timeliness of all other vaccine doses recommended by age 1 y (number of days delayed)	Other	Vaccination cards
Coverage of all vaccine doses recommended by age 1 y	Other	Vaccination cards

### Study Procedures

#### Cluster Randomization

In each district, all eligible health facilities will be assigned random IDs. Random IDs will be generated using a random number generator in Stata (version ≥16; StataCorp LLC) with a fixed seed to ensure reproducibility. In each district, the 10 clusters with the smallest random IDs will be allocated to either the early onset arm (odd ranks) or delayed onset arm (even ranks). Facilities that are not willing to participate or are deemed ineligible will be replaced with the next odd- or even-ranked facility, respectively. If the distributions of key characteristics of clusters (eg, the number of pregnant women/y, the number of births/y, the percentage of home births, vaccination coverage, and the number of days vaccinations are offered each wk) differ significantly between study arms, a covariate-constrained randomization [21,22] approach will be used to maximize the balance of these characteristics across study arms.

#### Blinding

Owing to the nature of the intervention, there will be no blinding of investigators, implementers (CHWs), data collectors, or participants.

#### Enrollment

Participants may be enrolled at their homes, health facilities, or other mutually agreed-upon locations. The potential risks and benefits of research participation will be carefully explained during the informed consent process (refer to the *Ethical Considerations* subsection). To maximize the reach of the intervention, participants without mobile phones will be able to designate a vaccine advocate of their choice to receive mobile phone–based messages.

#### Retention

Retention only applies to the prospective cohort. To ensure the retention of study participants, extensive contact information, including the names and mobile phone numbers of both parents, vaccine advocates, and other contacts, as well as expectations for the place of delivery, travel plans after birth, and GPS coordinates of homes will be documented at enrollment. In the event of child death, maternal death, or participant withdrawal, intervention activities related to the mother-child dyad will be suspended, and the dyad will be withdrawn from the study. In the event of child relocation or family travel during study activities, efforts will be made to collect outcomes data for the child using a combination of mobile phone–based surveys; a review of the child’s records at the local health facility; and proxy reports from the father, other legal guardians, or other informants. As the intervention involves local CHWs, for participants relocating outside their cluster for the remainder of the study period, intervention activities will stop after relocation.

### Data Collection

#### Overview

Trained research assistants will conduct enrollment and data collection in Kiswahili. Survey instruments will be developed in English, translated into Kiswahili, and back-translated into English. The development of the surveys will be informed by prior surveys [6,8].

#### Enrollment Survey

All participants will complete an enrollment survey. The survey will last approximately 45 minutes and assess knowledge and attitudes, sociodemographic characteristics of participants and their households, access barriers, digital literacy, residual knowledge gaps, and other correlates of vaccine uptake and timeliness. The mother’s engagement in prenatal care will be assessed via self-report and scanned prenatal care cards. For retrospective participants, the enrollment survey will also assess vaccination uptake and timeliness (Table 3), whereas for prospective participants, extensive contact information will be collected (refer to the *Retention* subsection).

#### Follow-Up Survey

Prospective participants will complete a follow-up survey 12 to 15 months after their child’s birth. The survey will last approximately 30 minutes. The primary purpose of the follow-up survey is to collect data on vaccination outcomes (Table 3). The follow-up survey will include the knowledge intervention that addresses each woman’s vaccination-specific knowledge gaps and assess vaccination attitudes and experiences with vaccine uptake and timeliness during the study period. The follow-up survey will also be used to update information on mothers’ engagement in prenatal care via self-report and scanned prenatal care cards. For intervention group women, the follow-up survey will also assess implementation measures, experiences with the intervention, and the acceptability and perceived efficacy of Chanjo Kwa Wakati.

#### Facility Survey

During the enrollment and follow-up work, we will conduct a survey of the health facility in each cluster. The purpose of the facility survey is to assess the implementation context. The survey will collect service information (eg, information on vaccine stockouts and facility closures), procedural knowledge (eg, vaccination dates, scheduling, and rescheduling), and contact information.

#### Qualitative Data

In-depth interviews (IDIs) will elucidate wide-ranging feedback on all constructs of the RE-AIM framework. IDIs will be conducted by local trained study staff in English or Kiswahili. Study staff will follow a semistructured interview guide. IDIs will be recorded to facilitate transcription and data analysis. Depending on the availability of the participants, IDIs may be conducted by phone or in person. IDIs are expected to last 45 to 60 minutes.

### Statistical Power

The aim 1 cluster randomized trial will include 1200 women, enrolled from 40 different clusters, across 3 distinct enrollment phases, with 10 women enrolled from each cluster in each phase. This equates to k=120 statistical clusters with n=10 women per cluster. Half of these clusters (k=60/120, 50%; n=600/1200, 50%) will be allocated to the intervention arm and half to the control arm. Assuming that the primary outcome—the timeliness of the Penta3 vaccine—has an SD of 28 days, the power of the trial to detect an intervention-related difference of 7 days is 0.95 with an intracluster correlation coefficient of 0.05; the power is 0.88 with an intracluster correlation coefficient of 0.10.

The aim 2 qualitative formative work will yield data from IDIs with key informants (n=12), facility-based health workers (up to n=40), CHWs (up to n=80), and mothers (up to n=60). Prior research suggests that thematic saturation can be achieved with 12 interviews, with metathemes presenting as early as 6 interviews [23-25]. Thus, these proposed sample sizes should be sufficient to achieve thematic saturation of implementation barriers and residual barriers to timely and equitable vaccinations, as well as to identify any regional variation in reasons for delayed vaccinations.

### Data Analysis

#### Quantitative Analysis of Intervention Effectiveness

The primary outcome measure of interest will be a continuous measure of vaccination timeliness, expressed as the delay, in days, between the vaccination due date and the date on which the vaccination was received for the third dose of the pentavalent vaccine, Penta3, due at age 14 weeks (Table 1). The secondary and other outcome measures include the timeliness and coverage of all vaccine doses recommended by age 1 year. Owing to the randomization, a comparison of mean outcomes between participants in the intervention group and those in the nonintervention group yields an unbiased estimate of the effect of the intervention.

To control for variation in sociodemographic characteristics across participants, seasonal effects, and other potential correlates of vaccination timeliness, outcomes will also be analyzed in a multivariable regression framework using accelerated failure time models with the completed vaccination dose as the failure event, age in days describing time to failure, and membership in the intervention versus control groups as the primary covariate of interest.

Robust SEs will be estimated to account for the clustering of observations across mother-child dyads enrolled from the same geographic clusters at the same time.

#### Analysis of Implementation Outcomes

We will generate summary measures (eg, means) for continuous data and use proportions to summarize categorical data. For outcomes assessed using validated measures (eg, the Acceptability of Intervention Measure [26]), we will present composite scale scores. Qualitative implementation outcomes will be analyzed as described below.

#### Analysis of Qualitative Data and Integration With Quantitative Data

Thematic analyses will be facilitated by qualitative software (eg, NVivo [Lumivero]) and a codebook made up of a priori and emergent structural codes based on the interview guide and 4 interrelated steps: reading, coding, data display, and data reduction [27]. The data will be used to fully map the Chanjo Kwa Wakati process from the perspectives of the mother and the CHW and characterize the opportunities and limitations of using digital and in-person resources to support vaccination timeliness. Using convergent mixed methods, we will use joint displays and narrative integration to connect the quantitative and qualitative data [28-30].

#### Analysis of Systematic Variation in Effectiveness

Extensive sensitivity analysis for the aim 1 primary and secondary outcomes analyses will characterize variation in intervention effectiveness with population, setting, and implementation factors. Variation in intervention effectiveness will be evaluated using interactions between geographic, maternal, or child characteristics and the intervention variable. Statistically significant parameters on interaction terms are indicative of differential intervention effects for different subgroups. To account for variation in implementation, sensitivity analysis will include per-protocol (as-treated) and intention-to-treat analyses in which a vector of variables describing the fidelity of the different intervention components will be used in place of the binary intervention variable.

#### Missing Data

The *Study Limitations and Adaptations* subsection in the *Discussion* section discusses considerations regarding missing data on outcomes and covariates and their handling in the analyses.

### Ethical Considerations

The study protocol, informed consent forms, and procedures have been reviewed and approved by the Health Sciences South Carolina Institutional Review Board (the reviewing institutional review board [IRB]; Pro00120675) and the ethics review committee of the National Institute for Medical Research in Tanzania (NIMR/HQ/R.8a/Vol.IX/4242), as well as the relying IRBs at Duke University (Pro00111772), Emory University (STUDY00005518), and the University of North Carolina at Chapel Hill. Informed consent will be sought from all study participants.

To maintain confidentiality, interviewers will conduct the consent process in a private space or in a mutually agreed-upon location where other people are not present. The interviewers will read out the consent form to potential participants and answer any questions. The potential risks and benefits of participation as well as the details of data confidentiality and use will be carefully explained in culturally appropriate and understandable language. Participants can refuse to participate and will only be consented after the research staff is satisfied that they understand all substantive provisions of the informed consent document. Persons with obvious psychological or psychiatric disorders that preclude informed consent will be excluded. Participants will be asked to provide written consent. They will be provided with a copy of the consent form. Informed consent documents will be developed in English, translated into Kiswahili, and back-translated into English.

Participants in the trial will be aged ≥15 years and either pregnant in their last trimester of pregnancy or mothers or legal guardians of children aged 12 to 23 months. For adolescents aged 15 to 17 years who are pregnant or mothers of children aged 12 to 23 months and are otherwise eligible to participate in the study, we obtained a waiver of parental consent. Tanzanian national policy indicates that adolescents aged <18 years who are sexually active and pregnant have the right to access reproductive health services (eg, HIV testing, care, and treatment [31], as well as family planning [32]) without parental or spousal consent, and they can make associated medical decisions on their own behalf. Given that these adolescents have this *adult* right to medical decision-making, we posit that they are also able to consent to research participation for themselves without parental or spousal consent. Risks from participation in this study are considered commensurate with ordinary daily life.

Protocol amendments will be submitted to the relevant IRBs for approval before implementation. Adverse events will be reported to the ethics review committee of the National Institute for Medical Research in Tanzania and the US-based IRBs.

Participants will be compensated for the time they spend participating in study activities. Ethically and culturally appropriate compensation will be established in collaboration with community advisers and other key informants. Subject to IRB approval and discussions with key informants, incentive amounts may be adjusted during the study period to account for exchange rate fluctuations and cost-of-living increases.

## Results

Results are pending. The study was funded in August 2022. Data collection is expected to last from February 2024 to July 2027.

## Discussion

### Relevance

The Chanjo Kwa Wakati intervention and proposed evaluation are aligned with the strategic priorities of the Immunization Agenda 2030 and Tanzanian priorities for digital health and community health workforce development [33,34]. Specifically, our study builds on a Tanzanian digital health investment road map [35] to digitize health care data and is in line with Tanzania’s 2020 National Operational Guideline for Community-Based Health Care Services, which seeks to build a strong community health workforce for health promotion, including for child health [36]. The relevance of our study extends beyond rural Tanzania. The low-cost strategies developed and evaluated in this study are applicable to other rural contexts where some populations are less likely to be vaccinated, experience poor vaccine access, or express low confidence in vaccinations, and our study may inform the development and implementation of multipronged programs to promote timely vaccinations in such settings.

### Contributions to the Literature

Our study is expected to make several important contributions to the scientific literature on digital health and vaccination interventions. First, numerous studies have documented low timeliness of vaccinations for children from LMICs, with a disproportionate burden for rural children [4,6,37-41]. Our study is significant because it seeks to evaluate an intervention for promoting vaccination timeliness for rural children. Second, lay health care workers such as CHWs play a critical role in delivering health services in LMICs. Our study will use a hybrid digital and in-person approach and contribute evidence on effective and scalable strategies for supporting lay health workers with digital health tools to promote childhood vaccination equity. Third, although many studies have evaluated mobile phone–based reminders for promoting childhood vaccinations [10], evidence is lacking for community-based systems that target multifaceted barriers to vaccinations. Our study will contribute data on the effectiveness of a community-based digital health intervention to promote equitable and timely vaccination in LMICs. Fourth, research studies support the use of screening tools to identify vaccination knowledge gaps and deliver targeted interventions to combat vaccine hesitancy. Our study will integrate CHW outreach and mobile phone–based vaccination education to bridge gaps in vaccination knowledge, respond to parental concerns, and reduce hesitancy. Finally, our study will contribute evidence on strategies to bridge the *digital divide* in rural areas and the *gender gap* in mobile phone access by using a combined digital and in-person approach.

### Study Limitations and Adaptations

#### Unknown Number of Eligible Women per Cluster

Although we estimate that 20 clusters per region are required to reach our enrollment targets for the cross-sectional and longitudinal samples, neither the number of eligible women per cluster nor the number of refusals are known a priori. If the sample size across clusters is too small, sampling may be extended to additional clusters until sample size targets have been reached in each region and study arm.

#### Randomization

If the distributions of key characteristics of clusters vary greatly between the initially specified study arms (based on randomized IDs), a covariate-constrained randomization approach may be used that maximizes the balance across study arms in the distribution of key characteristics of clusters.

#### Spillover and Contamination

Cluster randomization is expected to minimize spillover effects; however, contamination may exist for participants living at the boundaries between intervention and nonintervention clusters. Sensitivity analyses will evaluate potential spillover effects by including geographic proximity to an intervention cluster as a covariate for the nonintervention group women in our models.

#### External Events

A potential threat to validity stems from the possibility of a local vaccination campaign or selective stockouts among subsets of clusters during follow-up. Given our collaboration with regional and national stakeholders, enrollment from multiple clusters, multiple vaccination time points for each participant, and the robust design, the likelihood and potential impact of such external events on our findings is low. The effects of stockouts on timeliness will be assessed in the *per-protocol* sensitivity analysis.

#### Missing Data

If vaccination cards are missing for >10% of the cross-sectional sample, and their vaccination information cannot readily be ascertained from records at the local health facility, enrollment into the cross-sectional cohort will continue until key outcome measures can be ascertained for the target number of 400 mothers. This may include the expansion of enrollment to additional clusters. Enrollment into the longitudinal cohort will be increased proportionately. Missing data on other characteristics will be addressed using full information maximum likelihood estimation or sensitivity analyses [42-45].

#### Mobile Phone Access and Use

In the target population, mobile phone access and use is not universal, and mobile phones may be shared within households. Chanjo Kwa Wakati is specifically designed to compensate for gaps in mobile phone ownership and coverage, including through the designation of vaccine advocates who will receive reminders and in-person outreach by CHWs to those who are not reached by mobile phone.

#### Sustainability of Reminders and Incentives

Reminder messages and incentive offers, which were informed by our prior work, will be carefully reviewed by investigators and key informants to ensure their ethical implementation and sustainability for a scaled-up intervention.

#### Generalizability

To assess the generalizability of the study’s context and findings, study data and the relationships among key variables of interest will be compared with information in publicly available survey data and administrative data sources.

### Interpretation and Dissemination

If successful, this study will contribute data on the effectiveness and implementation of the Chanjo Kwa Wakati intervention that engages recent mothers to increase the timeliness and coverage of routine childhood vaccinations in rural Tanzania. Key findings will be presented in peer-reviewed manuscripts, presentations at national and international conferences, and media outlets. Policy and programmatic recommendations will be developed in collaboration with key informants and decision makers at the national, regional, and district levels.

## Appendix

Multimedia Appendix 1 CONSORT (Consolidated Standards of Reporting Trials) 2010 checklist of information to include when reporting a cluster randomized trial.

Multimedia Appendix 2 Peer-review report: Clinical Informatics and Digital Health Study Section, Healthcare Delivery and Methodologies Integrated Review Group, Center for Scientific Review, National Institutes of Health.

## Abbreviations

CHW: community health worker
CONSORT: Consolidated Standards of Reporting Trials
DPT: diphtheria-pertussis-tetanus
IDI: in-depth interview
IRB: institutional review board
LMICs: low- and middle-income countries
OPV: oral polio vaccine
RE-AIM: reach, effectiveness, adoption, implementation, and maintenance

## Data Availability

The data from this study will be available from the National Institute for Medical Research, Muhimbili Research Centre, Dar es Salaam, Tanzania, but restrictions apply that are governed by consent forms and data transfer agreements. Data may be made available by the authors upon reasonable request and with the permission of the National Medical Research Review Committee of the National Institute for Medical Research, Tanzania. The open-source digital health system will be made available to interested researchers and practitioners.
