# Supplementary material for: Leveraging Community Health Workers and a Responsive Digital Health System to Improve Vaccination Coverage and Timeliness in Resource-Limited Settings: Protocol for a Cluster Randomized Type 1 Effectiveness-Implementation Hybrid Study
Source: JMIR Res Protoc. 2024 Jan 12;13:e52523. doi: 10.2196/52523 (PMC10818232; doi:10.2196/52523)
Supplement: Multimedia Appendix 2 [file resprot_v13i1e52523_app2.pdf]

SUMMARY STATEMENT

PROGRAM CONTACT:
Alison Deckhut-Augustine
(301) 496-7551
adeckhut@niaid.nih.gov

(Privileged Communication)

Release Date: **02/28/2022**
Revised Date:

Application Number: **1 R01 AI170760-01**

Principal Investigators (Listed Alphabetically):

NGADAYA, ESTHER STANSLAUS
OSTERMANN, JAN (Contact)
VASUDEVAN, LAVANYA

Applicant Organization: UNIVERSITY OF SOUTH CAROLINA AT COLUMBIA

Review Group: **CIDH**
Clinical Informatics and Digital Health Study Section

Meeting Date: **02/09/2022**
Council: **MAY 2022**
Requested Start: **07/01/2022**

RFA/PA: **PA20-183**
PCC: **I2**
Dual IC(s): **HD**

Project Title: **"Chanjo Kwa Wakati" - Leveraging community health workers and a responsive digital health system to improve vaccination coverage and timeliness in rural settings**
SRG Action: **Impact Score:20 Percentile:2**
Next Steps: **Visit https://grants.nih.gov/grants/next_steps.htm**
Human Subjects: 30-Human subjects involved - Certified, no SRG concerns
Animal Subjects: 10-No live vertebrate animals involved for competing appl.
Gender: 1A-Both genders, scientifically acceptable
Minority: 5A-Only foreign subjects, scientifically acceptable
Age: 1A-Children, Adults, Older Adults, scientifically acceptable

Project Year	Direct Costs Requested	Estimated Total Cost
1	499,650	699,265
2	499,983	699,731
3	499,839	699,529
4	499,509	699,067
5	499,821	699,504
TOTAL	2,498,802	3,497,096

ADMINISTRATIVE BUDGET NOTE: The budget shown is the requested budget and has not been adjusted to reflect any recommendations made by reviewers. If an award is planned, the costs will be calculated by Institute grants management staff based on the recommendations outlined below in the COMMITTEE BUDGET RECOMMENDATIONS section.

1R01AI170760-01 Ostermann, Jan

RESUME AND SUMMARY OF DISCUSSION: This application proposes to conduct a cluster-randomized hybrid implementation trial to evaluate a multi-faceted intervention aimed at increasing utilization of vaccinations among mothers of infants in Tanzania. The significance of this proposal was driven by the innovative plan to utilize an integrated community-based digital health intervention to address vaccination inequities among rural and underserved populations in low-resource settings. During the discussion, the panel agreed that the rigor of the prior research is grounded in the scientific literature and provides a solid foundation for the focus on vaccination equities. The panel considered that the multiple PIs are well qualified with supplemental expertise and assembled an investigative team comprising the necessary complementary scientific expertise to ensure successful conduct of the proposed research. During the discussion, the reviewers also agreed that the study has several strengths such as, the use of strong theoretical framework, the strong preliminary data on the use of SMS and vaccination, the development of a software to increase health worker efficiency, the use of validated instruments, and the inclusion of an advisory group. The review panel also noted few minor and addressable weaknesses, including: the concern about the use of a no-intervention control group for the comparative study, limited clarity about the measures of cost-benefit analysis for Aim 2, a lack of consideration for sex as a biological variable for children, and the insufficient analytical details for Aims 1 and 3. During discussion the panel remained enthusiastic about this application’s innovation and significance. After discussion, the panel determined that the many strengths outweighed minor and highly addressable weaknesses and that the work would likely have a high impact on the field.

DESCRIPTION: Ensuring equitable vaccinations is critical for protecting all children against preventable and potentially dangerous infections such as polio, diphtheria, and measles. Yet, numerous studies have highlighted low vaccination coverage and timeliness, particularly among children from resource-limited settings. For example, in Tanzania, only 68% of children receive all basic vaccines that are recommended in the first year of life. Reasons for vaccination inequities are multifaceted; they include low caregiver knowledge about vaccines, and challenges with health service delivery and access. Health service interruptions during the global COVID-19 pandemic have further restricted opportunities for caregiver education, impacted vaccine access, and exacerbated vaccination inequities. Approaches that optimally utilize limited health workforce capacity and rapidly evolving digital health capacity for remote healthcare in resource-limited settings hold great potential for mitigating childhood vaccination inequities. We recently completed (1) a Fogarty-funded study (R21TW010262) that demonstrated the feasibility and efficacy of mobile phone-based reminders and conditional financial incentives for improving the coverage and timeliness of childhood vaccinations, and (2) a community health worker (CHW) intervention that was shown to be acceptable for mitigating caregiver knowledge gaps about childhood vaccines. Building on this prior work and with support from Tanzania’s National Immunization and Vaccine Development program, we propose to evaluate an integrated, community-based, digital intervention for promoting equity in childhood vaccinations. The outreach and educational intervention, called ”Chanjo Kwa Wakati” (“timely vaccination”), is targeted toward recent mothers and comprises a combination of CHW outreach and low-cost digital strategies (autonomous mobile phone-based vaccination promotion messages, reminders, stockout notifications, and incentive offers for timely vaccinations). In Aim 1, we will evaluate the effectiveness of Chanjo Kwa Wakati in promoting the coverage and timeliness of childhood vaccinations in a Type I effectiveness implementation hybrid trial. The trial will involve the staggered implementation of the intervention across catchment areas of 40 rural health facilities in two predominantly rural regions of Tanzania with large numbers of un- or under-vaccinated children. Vaccination outcomes will be analyzed for children born to 1200 women participating in the trial. In Aim 2, we will evaluate implementation factors associated with variations in intervention effectiveness, analyze the cost effectiveness of the intervention, and develop an implementation blueprint to guide scale-up to other settings. In Aim 3, we will evaluate the feasibility

and potential efficacy of a machine learning approach for proactively identifying children at risk of non- or delayed vaccinations and validate predictive models using vaccination data gathered in Aim 1. Study findings will inform future implementations and scale up of Chanjo Kwa Wakati, including potential interventions to improve vaccination equity for children living in rural, resource- limited, or underserved communities in the United States.

PUBLIC HEALTH RELEVANCE: Many children living in rural and resource-limited settings receive delayed or no vaccinations, placing them at risk for potentially dangerous vaccine-preventable diseases. The goal of the proposed research is to evaluate whether an integrated community-based digital health intervention can promote equitable routine vaccinations for children. The study will be conducted in rural Tanzania; however, findings may be broadly relevant for identifying digital health strategies that can be implemented by nursing or other non-physician cadres of health professionals, to bridge vaccination inequities among rural and underserved populations in the United States.

CRITIQUE 1

Significance: 1
Investigator(s): 2
Innovation: 2
Approach: 2
Environment: 1

Overall Impact: The proposed project is designed to increase vaccine effectiveness by expanding access to populations with low vaccination rates while also increasing compliance within the population. The goal is the design of a digital health intervention for new mothers to improve vaccination timeliness/rates, with a focus on rural LMIC communities. The aims focus on the development and evaluation of the intervention, and an exploratory aim focused on developing predictive models for children at risk for delayed vaccinations. The work is built on a strong theoretical foundation and prior work by the investigative team on the use of SMS and vaccination (albeit focused on a different health condition). The research team has some prior collaboration, and complementary expertise in the necessary domains of digital health, cultural competency, health economics, community based medicine, and qualitative/quantitative analyses. The research is also novel, and adapts methodologies developed for a different SES population to a rural, LMIC setting by combining the assets of community health workers and mHealth devices. The environment is also quite strong; there are domestic (US) and local (Tanzania) resources that maximize the effectiveness of international collaboration, and include the necessary computational, health, and administrative resources to coordinate the study. There is, however, concern about the use of a no-intervention control group for the comparative study; not providing any information to this group may not be ethically justified. Overall, the strong theoretical framework combined with an excellent team, novel approach, and research environment, suggest a high probability of success for the proposed work.

1. Significance:

Strengths

- PI presents rigorous prior research regarding the use of SMS with vaccine appointments.
- If successful, the work may lead to increased vaccine equity (e.g., rural, low income areas), increased vax timeliness, and the extension of health care to rural settings using dig. Technologies/mhealth.

- Prior work: experience with SMS based intervention (different population: HIV) in target country; some knowledge of factors limiting rural vaccination; validation of current intervention with a small subset of women (GOOD); validation of phone use in the target population; validation of digital reminders in target population. GOOD FEASIBILITY

Weaknesses

- None noted by reviewer

2. Investigator(s):

Strengths

- Research team has extensive prior experience with the complementary domains of cultural, behavioral, digital health; health economics, vaccine administration experience; qual/quant methods; community-based interventions; predictive analytics; and cost effectiveness in LMICs.
- Some members of the research team have prior research overlap and funded collaborations.
- There is appropriate infrastructure for the clinical trial, with administrative and organizational experience in the research team.

Weaknesses

- None noted by reviewer.

3. Innovation

Strengths

- The proposed approach mimics existing interventions designed for high income using combination of human + digital tools; this is a novel approach to adapting interventions designed for populations of different demographics.
- The potential generation of LMIC specific ML algorithms (and data that can be used by others for model training) is a novel contribution to science regarding vaccination responses in similar populations.
- The novel research design is optimized to simultaneously measure effectiveness, combine qual/quant information, and to use a validated implementation framework (RE-AIM) to maximize eventual translation to practice.

Weaknesses

- None noted by reviewer.

4. Approach:

Strengths

- The proposed intervention is built on a validated RE-AIM framework, increasing probability of success. Additionally, the software framework uses validated, open technologies.
- The to be developed software increases health worker efficiency by automating some tasks, minimizing CHW burden.
- The qualitative/self-report instrument has already been validated with target population

- Inclusion of an advisory group maximizes cultural appropriateness and reduces risk of failure due to misunderstanding between the research team and participants.

Weaknesses

- The use of a no intervention control group may not be appropriate. Not providing even a minimum of materials or information may not be ethically justifiable.

5. Environment:

Strengths

- The research environment is excellent. The domestic (US) environment includes the necessary analytical resources, as well as global/international research resources. The LMIC partner includes a research institute with well aligned mission and strong organizational structure.
- Given this is a clinical trial, the presented research infrastructure (administrative, testing centers, etc.) are appropriate. This is based on prior experience of the research team and partner organizations in the LMIC.

Weaknesses

- None noted by reviewer.

Study Timeline:

Strengths

- Timeline allots sufficient time for human subjects recruitment and intervention components.

Weaknesses

- Model evaluation period (Aim 3) may not be sufficient (only 2 months per site).

Protections for Human Subjects:

Acceptable Risks and/or Adequate Protections

- Risks are appropriately communicated, perhaps reflecting prior experience of investigators with target population.

Data and Safety Monitoring Plan (Applicable for Clinical Trials Only):

Acceptable

  - Plan is acceptable, though no DSMB or named study monitor is included.

Inclusion Plans:

- Sex/Gender: Distribution justified scientifically
- Race/Ethnicity: Distribution justified scientifically
- For NIH-Defined Phase III trials, Plans for valid design and analysis: Not applicable
- Inclusion/Exclusion Based on Age: Distribution justified scientifically
- Distributions based on target population (pregnant women) and demographics of target location (Tanzania).

Vertebrate Animals:
Not Applicable (No Vertebrate Animals)

Biohazards:
Not Applicable (No Biohazards)

Applications from Foreign Organizations:
None noted by reviewer.

Resource Sharing Plans:
Acceptable

Budget and Period of Support:
Recommend as Requested

CRITIQUE 2

Significance: 2
Investigator(s): 2
Innovation: 2
Approach: 3
Environment: 1

Overall Impact: This is a multi-PI application. The application proposes to increase vaccination in low-income settings (Tanzania) through low-cost digital strategies. The application is a clinical trial. The study team has extensive experience working with investigators and the population in Tanzania. The proposed project is seen as significant and the rigor of the prior research is strong. Successful completion of the proposed project could broadly impact rural vaccination rates. The team is well-suited to perform the proposed research and has a rich collaborative and multinational experience. There are some minor concerns with not including a biostatistician on the design of the statistical analysis. The project is seen as innovative although new methods are not being applied, they are implemented in novel ways. The approach is robust and aims 1 and 2 are well-described and sound. There are some concerns with the machine learning in the exploratory aim 3. It does not take into account potential generalizability to other populations outside the one provided. It is not clear what data elements will be included and while stakeholders will be queried, there is no planned time for this and no design for including them in model development. There are some minor concerns about the resulting models being overfit to the provided population. Overall, there is high enthusiasm for the proposal and it is likely to result in a successful intervention, increased vaccinations, and an extensible method to improve outreach in rural areas.

1. Significance:

Strengths

- The rigor of the prior research is strong and well described.

- The rationale for a clinical trial is well-supported and the study team has the necessary expertise and experience.
- Increasing vaccination rates in children and in low-income settings is seen as significance and important.
- Digital health interventions have been shown to increase vaccination rates and applying these in low income and rural settings is seen as important.
- Successful completion of the project could help to improve the vaccination rates and health of children in all rural settings with parental access to cellular phones

Weaknesses

- A clinical trial is not required to test the safety of vaccinations but the proposed study is appropriate in testing the efficacy of the intervention to increase vaccinations.

2. Investigator(s):

Strengths

- The study team has the necessary expertise in health policy, immunization demand, epidemiology, implementation science, data mining and data integration, and economics.
- This is a mPI application.
- The study team has extensive experience working with and in Tanzania. The team has previously collaborated.

Weaknesses

- The lack of a biostatistician is a concern as it relates to the proposed analysis of the intervention.

3. Innovation:

Strengths

- The proposed interventions are not novel, but are being applied in a novel setting and new ways.
- The integration of machine learning for prediction in low income settings could potentially shift clinical practice for targeting vaccinations.

Weaknesses

- The implementation framework proposed is not seen as novel, but is seen as important and a robust study design.

4. Approach:

Strengths

- The team has already successfully completed an R21 Fogarty award in Tanzania.
- The preliminary work is appropriate and robust.
- The aim 1 clinical trial to increase vaccination is well-described and has appropriate strategies and analysis in place.

- The scientific rigor of aims 1 and 2 is strong and well-described.
- The project will establish feasibility of a large-scale, mobile-phone based vaccination strategy.
- Aim 3 is well-described and the machine learning on existing data is sound.

Weaknesses

- Sex as a biological variable is taken into account with mothers and but not handled with the children and should be taken into account in the resulting model.
- It is not clear who will be performing the cost-effectiveness analysis in aim 2.
- Aims 2 and 3 are dependent on the successful clinical trial in aim 1 but this is seen as a minor concern given the experience of the study team.
- Aim 3 does not take into account potential generalizability to other populations outside the one provided. It is not clear what data elements will be included and while stakeholders will be queried, there is no planned time for this and no design for including them in model development. There are some minor concerns about the resulting models being overfit to the provided population.

5. Environment:

Strengths

- The University of South Carolina has experience with international research and appropriate resources for computing.
- Duke University is outstanding and has participated and collaborated in the past.
- UNC Chapel Hill school of social work is excellent.
- National Institute for medical Research has the necessary medical and research capabilities for the proposed project.

Weaknesses

- None noted by reviewer.

Study Timeline:

Strengths

- Timeline is acceptable for proposed study

Weaknesses

- Site visits are not listed on the timeline

Protections for Human Subjects:

Acceptable Risks and/or Adequate Protections

Data and Safety Monitoring Plan (Applicable for Clinical Trials Only):

Acceptable

Inclusion Plans:

- Sex/Gender: Distribution justified scientifically

- Race/Ethnicity: Distribution justified scientifically
- For NIH-Defined Phase III trials, Plans for valid design and analysis: Not applicable
- Inclusion/Exclusion Based on Age: Distribution justified scientifically

Vertebrate Animals:
Not Applicable (No Vertebrate Animals)

Biohazards:
Not Applicable (No Biohazards)

Applications from Foreign Organizations:
Justified

Resource Sharing Plans:
Acceptable

- It would be better to see the mParis platform shared in a way that is more easily accessible.

Budget and Period of Support:
Recommend as Requested

CRITIQUE 3

Significance: 1
Investigator(s): 1
Innovation: 3
Approach: 3
Environment: 1

Overall Impact: Dr. Ostermann and colleagues propose to evaluate a multi-faceted intervention, (“Chanjo Kwa Wakati”, including community health worker (CHW) outreach and variety of automated informatics interventions) aimed at increasing utilization of vaccinations among mothers of infants (<1yr) in Tanzania. The project would include a cluster-randomized hybrid (CHW + informatics) implementation trial of 1200 mother-child dyads across 40 sites, and then evaluate effectiveness and cost-effectiveness of the intervention as compared to standard practice. Later in the project the team would then use machine learning techniques to develop a model predicting risk of non- or delayed vaccinations, based on the premise that risk-tailored approaches could optimize effectiveness and cost-effectiveness in resource-constrained environments. In general, the intervention design is interesting and creative, the study design is rigorous, and the work would lead to generalizable knowledge on how to effectively deploy clinical informatics to address socioeconomic and other health disparities (not just within vaccination). High enthusiasm for these aspects was attenuated by lack of clarity on details of the analysis approaches in Aims 1 and 3.

1. Significance:

Strengths

- Globally, only 86% of children under age 5 are immunized in timely fashion, and lack of immunization disproportionately impacts low-resource populations.
- Vaccination against disease is one of the most effective ways to increase life expectancy in low- and middle-income countries.
- Progress on improving vaccination coverage has stalled over the last 10 years.
- Implementation of pediatric vaccination is complicated by disparities in preventive health care access/utilization (e.g., across the metro/rural divide and or digital divide) as well as differences in parental attitudes and perspectives toward vaccination.
- Vaccination delays are more pronounced in rural settings; as technology access and use steadily increases, the work would take advantage of new opportunities for reaching this challenging demographic via a combination of HCW outreach and digital interventions.
- Health care delivery-based efforts to address vaccine hesitancy in LMICs are scant, despite evidence of their effectiveness in high-income countries.

Weaknesses

- None noted by reviewer.

2. Investigator(s):

Strengths

- Team has extensive experience and prior collaborations on NIH-funded and other federally funded research.
- The international research team and the proposal present a transdisciplinary approach to the problem of vaccination coverage in LIMCs.
- PI has successfully conducted research in Tanzania, which is the setting of the proposed intervention.

Weaknesses

- None noted by reviewer.

3. Innovation:

Strengths

- Focused approach to addressing barriers and inequities in digital health technology (DHT) access/use through proactive DHT development.
- Focusing on new mothers (whose children are <1 year of age) aligns with vaccination guidelines and could maximize effectiveness of interventions to increase vaccination utilization.
- Combination of HCW involvement and DHT may particularly maximize effectiveness in low-socioeconomic position populations.

Weaknesses

- Proposed machine learning approaches are routine (“off-the-shelf”) and not innovative.

4. Approach:

Strengths

- Choice of primary outcome for the cluster-randomized hybrid implementation trial (Aim 1), i.e., time from vaccination due date to receipt, is desirable and appropriate.
- Organization of the prospective data collection to facilitate validation of predictive models derived externally (Aim 3) is clever.

Weaknesses

- Details on the difference-in-differences analysis approach as contextualized to the survival analysis framework are unclear. Specifically, the explicit quantities derived from the model to characterize treatment effect are in the form of regression coefficients. While relative assessments of treatment outcomes are not irrelevant, this fails to capture absolute assessments of vaccination rates, which are the basis of the proposal.
- The selected measure of benefit in the proposed cost-benefit analysis of Aim 2 – namely, the time after vaccination due date at which 80% of a population has been vaccinated – may be sub-optimal for measuring cost-benefit of this public health intervention, since this measure would not sufficiently capture longer-term effects of overall increases in vaccination coverage. (This is not to say that the expressed measure is unimportant.)
- Aim 3 modeling methods are vague and inadequately contextualized to the specific modeling task. For example, the approach lists “off-the-shelf” methods without considering their appropriateness for the task of predicting vaccination uptake or delayed vaccination or their relative benefits over alternative approaches for the task.
- It is unclear how the long short-term memory neural network model would apply to the proposed cross-sectional prediction tasks.
- The decision problem to be informed by prediction modeling/machine learning is not formally defined, nor are the aspects of predictive performance that would be most relevant to the decision problem.

5. Environment:

Strengths

- Inter-institutional collaboration (U South Carolina, Duke University, Tanzania National Institute for Medical Research) is supported through previous long-term collaboration.
- Project plan includes regular site visits of key personnel to Tanzania.
- Scientific resources at participating institutions are suitable.

Weaknesses

- None noted by reviewer.

Study Timeline:

Strengths

- Detailed and organized. Preparatory activities will be completed within the first year, with the prospective study being carried out between late Year 1 and early Year 5.

Weaknesses

- Cost effectiveness analyses in Aim 2 planned to begin in year 5; it is unclear whether components of this analysis should be initiated prior to the completion of the study intervention, with the concern being that cost-effectiveness analyses are time- and resource-intensive and 6-9 months in Year 5 may be insufficient time to complete the aim.
- Cyclic approach to Aim 3 (annual sets of activities) does not clearly align with the described approach in the research plan.

Protections for Human Subjects:

Acceptable Risks and/or Adequate Protections

- Special consideration is given to children aged 16-17 years, who, in Tanzania, are legally permitted to make medical decisions relating to reproductive health. Owing to the 25% rate of pregnancy/delivery among females aged <18 years in Tanzania, the team would request a waiver of parental consent from IRBs at USC and Duke and in Tanzania to include these children if they are otherwise eligible to participate in the study. This is on the basis that the planned interventions are not outside risks encountered during ordinary life. Steps are taken to protect against breaches of sensitive data per HIPAA requirements, recognize signs of emotional distress that are inconsistent with the ability to provide informed consent, and adverse reactions that might be the result of receiving unwanted SMS messages. The team would consult an ethicist to review and help the team adapt SMS and other technological interventions to minimize risks of these adverse outcomes and experiences.

Data and Safety Monitoring Plan (Applicable for Clinical Trials Only):

Acceptable

- Data will be de-identified and coded to unique study identifiers. Data will be transmitted only under established Data Transfer Agreements. Reporting of study results will only be made in aggregate. Any adverse events would be reported to the institutions' IRBs.

Inclusion Plans:

- Sex/Gender: Distribution justified scientifically
- Race/Ethnicity: Distribution justified scientifically
- For NIH-Defined Phase III trials, Plans for valid design and analysis: Scientifically acceptable
- Inclusion/Exclusion Based on Age: Distribution justified scientifically
- The study will be conducted among mother-infant dyads in Tanzania. It is expected that the study participants will be of African descent. The team has carefully planned for inclusion of mothers aged 16 years and above if deemed to be ethically acceptable (otherwise, 18 years and above) in order to be inclusive of mothers of child-bearing age.

Vertebrate Animals:

Not Applicable (No Vertebrate Animals)

Biohazards:

Not Applicable (No Biohazards)

Applications from Foreign Organizations:
None noted by reviewer.

Resource Sharing Plans:
Acceptable

- Study data will be made publicly available upon request to the PI after publication of findings.

Budget and Period of Support:
Recommend as Requested

THE FOLLOWING SECTIONS WERE PREPARED BY THE SCIENTIFIC REVIEW OFFICER TO SUMMARIZE THE OUTCOME OF DISCUSSIONS OF THE REVIEW COMMITTEE, OR REVIEWERS' WRITTEN CRITIQUES, ON THE FOLLOWING ISSUES:

PROTECTION OF HUMAN SUBJECTS: ACCEPTABLE

INCLUSION OF WOMEN PLAN: ACCEPTABLE

INCLUSION OF MINORITIES PLAN: ACCEPTABLE

INCLUSION ACROSS THE LIFESPAN: ACCEPTABLE

COMMITTEE BUDGET RECOMMENDATIONS: The budget was recommended as requested.

Footnotes for 1 R01 AI170760-01; PI Name: Ostermann, Jan

NIH has modified its policy regarding the receipt of resubmissions (amended applications).See Guide Notice NOT-OD-18-197 at https://grants.nih.gov/grants/guide/notice-files/NOT-OD-18-197.html. The impact/priority score is calculated after discussion of an application by averaging the overall scores (1-9) given by all voting reviewers on the committee and multiplying by 10. The criterion scores are submitted prior to the meeting by the individual reviewers assigned to an application, and are not discussed specifically at the review meeting or calculated into the overall impact score. Some applications also receive a percentile ranking. For details on the review process, see http://grants.nih.gov/grants/peer_review_process.htm#scoring.

MEETING ROSTER

Clinical Informatics and Digital Health Study Section
Healthcare Delivery and Methodologies Integrated Review Group
CENTER FOR SCIENTIFIC REVIEW
CIDH
02/09/2022 - 02/10/2022

Notice of NIH Policy to All Applicants: Meeting rosters are provided for information purposes only. Applicant investigators and institutional officials must not communicate directly with study section members about an application before or after the review. Failure to observe this policy will create a serious breach of integrity in the peer review process, and may lead to actions outlined in NOT-OD-14-073 at https://grants.nih.gov/grants/guide/notice-files/NOT-OD-14-073.html, NOT-OD-15-106 at https://grants.nih.gov/grants/guide/notice-files/NOT-OD-15-106.html, and NOT-OD-18-115 at https://grants.nih.gov/grants/guide/notice-files/NOT-OD-18-115.html, including removal of the application from immediate review.

CHAIRPERSON(S)

JAFARI, ROOZBEH, PHD
PROFESSOR
DEPARTMENTS OF BIOMEDICAL ENGINEERING,
COMPUTER SCIENCE AND ENGINEERING,
AND ELECTRICAL AND COMPUTER ENGINEERING
TEXAS A&M UNIVERSITY
COLLEGE STATION, TX 77843

MEMBERS

ABDELRAHMAN, SAMIR E, PHD *
ASSISTANT PROFESSOR
DEPARTMENT OF BIOMEDICAL INFORMATICS
UNIVERSITY OF UTAH
SALT LAKE, UT 84108

ALEMI, FARROKH, PHD *
PROFESSOR
DEPARTMENT OF HEALTH ADMINISTRATION AND POLICY
GEORGE MASON UNIVERSITY
FAIRFAX, VA 22030

ALPERN, ELIZABETH RACHEL, MD
PROFESSOR
DEPARTMENT OF PEDIATRICS
ANN AND ROBERT H. LURIE CHILDREN'S HOSPITAL
FEINBERG SCHOOL OF MEDICINE
NORTHWESTERN UNIVERSITY
CHICAGO, IL 60611

AUDU, MUSA L, PHD
PROFESSOR
DEPARTMENT OF BIOMEDICAL ENGINEERING
ADVANCED PLATFORM TECHNOLOGY CENTER
CASE WESTERN RESERVE UNIVERSITY
CLEVELAND, OH 44106

BASS, SARAH B, PHD *
ASSOCIATE PROFESSOR
DIRECTOR
RISK COMMUNICATION LABORATORY
TEMPLE UNIVERSITY
PHILADELPHIA, PA 19122

BELL, DOUGLAS SCOTT, MD, PHD *
PROFESSOR
DEPARTMENT OF MEDICINE
CLINICAL AND TRANSLATIONAL SCIENCE INSTITUTE
UNIVERSITY OF CALIFORNIA, LOS ANGELES
LOS ANGELES, CA 90222

BORSON, SOO, MD *
PROFESSOR
DEPARTMENT OF CLINICAL FAMILY MEDICINE
KECK SCHOOL OF MEDICINE
UNIVERSITY OF SOUTHERN CALIFORNIA
LOS ANGELES, CA 90033

CULLEY, JOAN MARIE, PHD
PROFESSOR EMERITA
COLLEGE OF NURSING
UNIVERSITY OF SOUTH CAROLINA
COLUMBIA, SC 29208

DALTON, JARROD, PHD *
ASSOCIATE STAFF
DEPARTMENT OF QUANTITATIVE HEALTH SCIENCES
CLEVELAND CLINIC FOUNDATION
CLEVELAND, OH 44195

DEFILIPPI, CHRISTOPHER R, MD *
VICE CHAIRMAN OF ACADEMIC AFFAIRS
INOVA HEART AND VASCULAR INSTITUTE
VIRGINIA, VA 22042

DEXHEIMER, JUDITH W, PHD
ASSOCIATE PROFESSOR
DEPARTMENT OF PEDIATRICS
AND BIOMEDICAL INFORMATICS
CINCINNATI CHILDREN'S HOSPITAL MEDICAL CENTER
UNIVERSITY OF CINCINNATI
CINCINNATI, OH 45229

DUNN LOPEZ, KAREN, PHD *
ASSOCIATE PROFESSOR
CENTER FOR NURSING CLASSIFICATION
AND CLINICAL EFFECTIVENESS
COLLEGE OF NURSING
THE UNIVERSITY OF IOWA
IOWA CITY, IA 52242

EDEN, KAREN BEEKMAN, PHD *
PROFESSOR
DEPARTMENT OF MEDICAL INFORMATICS
AND CLINICAL EPIDEMIOLOGY
OREGON HEALTH AND SCIENCE UNIVERSITY
PORTLAND, OR 97239

GAJIC, OGNJEN, MD *
PROFESSOR
DEPARTMENT OF MEDICINE
COLLEGE OF MEDICINE
MAYO CLINIC, ROCHESTER
ROCHESTER, MN 55905

JARRÍN MONTANER, OLGA F., PHD *
ASSISTANT PROFESSOR
DIVISION OF NURSING SCIENCE
RUTGERS SCHOOL OF NURSING
RUTGERS UNIVERSITY
NEW BRUNSWICK, NJ 08901

JORDAN, JENNIFER HAWTHORNE, PHD *
ASSISTANT PROFESSOR
DEPARTMENT OF BIOMEDICAL ENGINEERING
VIRGINIA COMMONWEALTH UNIVERSITY
RICHMOND, VA 23284

KHAIRAT, SAIF, PHD *
ASSOCIATE PROFESSOR
SCHOOL OF NURSING
CAROLINA HEALTH INFORMATICS PROGRAM (CHIP)
UNIVERSITY OF NORTH CAROLINA
CHAPEL HILL, NC 27514

KHOKHAR, ASHFAQ, PHD *
PROFESSOR
DEPARTMENT OF ELECTRICAL AND COMPUTER
ENGINEERING
IOWA STATE UNIVERSITY
AMES, IA 50011

KIRKENDALL, ERIC STEVEN, MD *
PROFESSOR
DEPARTMENT OF PEDIATRICS
DIRECTOR OF DIGITAL HEALTH INNOVATION
WAKE FOREST UNIVERSITY
WINSTON-SALEM, NC 27109

LAKSHMINARAYAN, KAMAKSHI, PHD *
ASSOCIATE PROFESSOR
DEPARTMENT OF NEUROLOGY, MEDICAL SCHOOL
DIVISION OF EPIDEMIOLOGY & COMMUNITY HEALTH
SCHOOL OF PUBLIC HEALTH
UNIVERSITY OF MINNESOTA
MINNEAPOLIS, MN 55454

LIU, YANXI, PHD *
PROFESSOR
DEPARTMENT OF COMPUTER SCIENCE
AND ENGINEERING
PENNSYLVANIA STATE UNIVERSITY
UNIVERSITY PARK, PA 16802

M'KOMA, AMOSY EPHREIM, MD, PHD *
ASSOCIATE PROFESSOR
DEPARTMENT OF BIOCHEMISTRY AND CANCER BIOLOGY
DIVISION OF BIOMEDICAL SCIENCES
MEHARRY MEDICAL COLLEGE SCHOOL OF MEDICINE
NASHVILLE, TN 37208

NADKARNI, GIRISH NITIN, MD *
ASSOCIATE PROFESSOR
DEPARTMENT OF MEDICINE, NEPHROLOGY
ICAN SCHOOL OF MEDICINE AT MT SINAI
NEW YORK, NY 10029

NEMATI, SHAMIM, PHD *
ASSISTANT PROFESSOR
DEPARTMENT OF BIOMEDICAL INFORMATICS
UNIVESITY OF CALIFORNIA, SAN DIEGO
LA JOLLA, CA 92093

PARIKH, RAVI BHARAT, MD *
ASSISTANT PROFESSOR
DEPARTMENT OF MEDICAL ETHICS AND HEALTH POLICY
DEPARTMENT OF MEDICINE
PERELMAN SCHOOL OF MEDICINE
UNIVERSITY OF PENNSYLVANIA
PHILADELPHIA, PA 19104

PELPHREY, KEVIN A, PHD *
HARRISON-WOOD JEFFERSON SCHOLARS FOUNDATION
PROFESSOR OF NEUROLOGY
DEPARTMENT OF NEUROLOGY
UNIVERSITY OF VIRGINIA
CHARLOTTESVILLE, VA 22908

PURNELL, TANJALA, PHD *
ASSISTANT PROFESSOR
DEPARTMENT OF EPIDEMIOLOGY
JOHNS HOPKINS UNIVERSITY
BALTIMORE, MD 21205

REHG, JAMES M, PHD
PROFESSOR
CENTER FOR BEHAVIORAL IMAGING
SCHOOL OF INTERACTIVE COMPUTING
GEORGIA INSTITUTE OF TECHNOLOGY
ATLANTA, GA 30332

ROBERTS, KIRK E, PHD *
ASSOCIATE PROFESSOR
SCHOOL OF BIOMEDICAL INFORMATICS
UNIVERSITY OF TEXAS – HOUSTON
HOUSTON, TX 77030

SJODING, MICHAEL WILLIAM, MD *
ASSISTANT PROFESSOR
UNIVERSITY OF MICHIGAN MEDICAL SCHOOL
UNIVERSITY OF MICHIGAN
ANN ARBOR, MI 48109

SKUBIC, MARJORIE, PHD
PROFESSOR
DEPARTMENT OF ELECTRICAL ENGINEERING
AND COMPUTER SCIENCE
UNIVERSITY OF MISSOURI
COLUMBIA, MO 65211

STAUDENMAYER, JOHN W, PHD *
PROFESSOR
DEPARTMENT OF MATHEMATICS AND STATISTICS
UNIVERSITY OF MASSACHUSETTS-AMHERST
AMHERST, MA 01003

SWARD, KATHERINE ANN, PHD
PROFESSOR
DEPARTMENT OF BIOMEDICAL INFORMATICS
COLLEGE OF NURSING
UNIVERSITY OF UTAH
SALT LAKE CITY, UT 84112

TANDON, ANIMESH, MD *
ASSOCIATE STAFF
DIRECTOR OF CARDIOVASCULAR INNOVATION
DEPARTMENT OF PEDIATRIC CARDIOLOGY
CLEVELAND CLINIC CHILDREN'S HOSPITAL
CLEVELAND, OH 44195

VAILLANCOURT, DAVID E, PHD *
PROFESSOR
DEPARTMENT OF APPLIED PHYSIOLOGY AND KINESIOLOGY
UNIVERSITY OF FLORIDA
GAINESVILLE, FL 32611

VRANCEANU, ANA-MARIA, PHD *
FOUNDER & DIRECTOR, IBHCRP AND ASSOCIATE PROFESSOR
DEPARTMENT OF PSYCHIATRY
MASS GENERAL HOSPITAL
BOSTON, MA 02476

WADE, ERIC, PHD
ASSOCIATE PROFESSOR
DEPARTMENT OF MECHANICAL, AEROSPACE
AND BIOMEDICAL ENGINEERING
UNIVERSITY OF TENNESSEE, KNOXVILLE
KNOXVILLE, TN 37996

WALJI, MUHAMMAD, PHD *
PROFESSOR
DEPARTMENT OF DIAGNOSTIC AND BIOMEDICAL SCIENCES
UNIVERSITY OF TEXAS HEALTH SCIENCE CENTER
AT HOUSTON
HOUSTON, TX 77030

WANG, DONGWEN, PHD *
PROFESSOR
DEPARTMENT OF BIOMEDICAL INFORMATICS
ARIZONA STATE UNIVERSITY
SCOTTSDALE, AZ 85259

YU, DENNY, PHD *
ASSISTANT PROFESSOR
INDUSTRIAL ENGINEERING
PURDUE UNIVERSITY
WEST LAFAYETTE, IN 47907

ZHU, VIVIENNE, MD *
ASSOCIATE PROFESSOR
DEPARTMENT OF PUBLIC HEALTH SCIENCES
MEDICAL UNIVERSITY OF SOUTH CAROLINA
CHARLESTON, SC 29425

MAIL REVIEWER(S)

SCHONBERGER, ROBERT, MD
ASSOCIATE PROFESSOR
DEPARTMENT OF ANESTHESIOLOGY
SCHOOL OF MEDICINE
YALE UNIVERSITY
NEW HAVEN, CT 06510

ZESIEWICZ, THERESA A, MD
PROFESSOR
DEPARTMENT OF NEUROLOGY
UNIVERSITY OF SOUTH FLORIDA
TAMPA, FL 33612

SCIENTIFIC REVIEW OFFICER

HEWETT-MARX, PAUL, PHD
SCIENTIFIC REVIEW OFFICER
CENTER FOR SCIENTIFIC REVIEW
NATIONAL INSTITUTE OF HEALTH
BETHESDA, MD 20892

EXTRAMURAL SUPPORT ASSISTANT

NJOKU, PHILIP C
EXTRAMURAL SUPPORT ASSISTANT
DIVISION OF AIDS, BEHAVORIAL, POPULATION SCIENCES
HEALTHCARE DELIVERY AND METHODOLOGIES (HDM)
NATIONAL INSTITUTES OF HEALTH
BETHESDA, MD 20892

* Temporary Member. For grant applications, temporary members may participate in the entire meeting or may review only selected applications as needed.

Consultants are required to absent themselves from the room during the review of any application if their presence would constitute or appear to constitute a conflict of interest.
